# Evaluation of the safety, immunogenicity, and faecal shedding of novel oral polio vaccine type 2 in healthy newborn infants in Bangladesh: a randomised, controlled, phase 2 clinical trial

**DOI:** 10.1016/S0140-6736(22)02397-2

**Published:** 2023-01-14

**Authors:** Khaelqu Zaman, Ananda S Bandyopadhyay, Masuma Hoque, Christopher Gast, Mohammad Yunus, Khondoker M Jamil, Bernardo A Mainou, Jennifer L Konopka-Anstadt, William S Hendley, Annelet Vincent, Ralf Clemens, Sue Ann Costa Clemens, Allen G Ross, John D Clemens, Erman Tritama

**Affiliations:** a International Centre for Diarrhoeal Disease Research, Chandpur, Bangladesh; b Bill & Melinda Gates Foundation, Seattle, WA, USA; c PATH, Seattle, WA, USA; d National Polio and Measles Laboratory, Institute of Public Health, Dhaka, Bangladesh; e US Centers for Disease Control and Prevention, Atlanta, GA, USA; f Global Research in Infectious Diseases, Rio de Janeiro, Brazil; g Department of Paediatrics, Oxford University, Oxford, UK; h Rural Health Research Institute, Charles Sturt University, Wagga Wagga, NSW, Australia; i International Vaccine Institute, Seoul, South Korea; j PT Bio Farma, Bandung, Indonesia

## Abstract

**Background:**

Type 2 circulating vaccine-derived polioviruses (cVDPV2) from Sabin oral poliovirus vaccines (OPVs) are the leading cause of poliomyelitis. A novel type 2 OPV (nOPV2) has been developed to be more genetically stable with similar tolerability and immunogenicity to that of Sabin type 2 vaccines to mitigate the risk of cVDPV2. We aimed to assess these aspects of nOPV2 in poliovirus vaccine-naive newborn infants.

**Methods:**

In this randomised, double-blind, controlled, phase 2 trial we enrolled newborn infants at the Matlab Health Research Centre, Chandpur, Bangladesh. We included infants who were healthy and were a single birth after at least 37 weeks' gestation. Infants were randomly assigned (2:1) to receive either two doses of nOPV2 or placebo, administered at age 0–3 days and at 4 weeks. Exclusion criteria included receipt of rotavirus or any other poliovirus vaccine, any infection or illness at the time of enrolment (vomiting, diarrhoea, or intolerance to liquids), diagnosis or suspicion of any immunodeficiency disorder in the infant or a close family member, or any contraindication for venipuncture. The primary safety outcome was safety and tolerability after one and two doses of nOPV2, given 4 weeks apart in poliovirus vaccine-naive newborn infants and the primary immunogenicity outcome was the seroconversion rate for neutralising antibodies against type 2 poliovirus, measured 28 days after the first and second vaccinations with nOPV2. Study staff recorded solicited and unsolicited adverse events after each dose during daily home visits for 7 days. Poliovirus neutralising antibody responses were measured in sera drawn at birth and at age 4 weeks and 8 weeks. This study is registered on ClinicalTrials.gov, NCT04693286.

**Findings:**

Between Sept 21, 2020, and Aug 16, 2021, we screened 334 newborn infants, of whom three (<1%) were found to be ineligible and one (<1%) was withdrawn by the parents; the remaining 330 (99%) infants were assigned to receive nOPV2 (n=220 [67%]) or placebo (n=110 [33%]). nOPV2 was well tolerated; 154 (70%) of 220 newborn infants in the nOPV2 group and 78 (71%) of 110 in the placebo group had solicited adverse events, which were all mild or moderate in severity. Severe unsolicited adverse events in 11 (5%) vaccine recipients and five (5%) placebo recipients were considered unrelated to vaccination. 306 (93%) of 330 infants had seroprotective maternal antibodies against type 2 poliovirus at birth, decreasing to 58 (56%) of 104 in the placebo group at 8 weeks. In the nOPV2 group 196 (90%) of 217 infants seroconverted by week 8 after two doses, when 214 (99%) had seroprotective antibodies.

**Interpretation:**

nOPV2 was well tolerated and immunogenic in newborn infants, with two doses, at birth and 4 weeks, resulting in almost 99% of infants having protective neutralising antibodies.

**Funding:**

Bill & Melinda Gates Foundation.

## Introduction

Although live oral poliovirus vaccines (OPVs) are safe and effective and have led to the elimination of poliomyelitis from most of the world, in rare circumstances the attenuated viruses in OPVs can mutate and reacquire neurovirulence. This mutation can result in vaccine-associated paralytic polio in vaccine recipients and susceptible close contacts and, in settings of persistently poor immunisation coverage, can lead to the emergence of circulating vaccine-derived polioviruses (cVDPVs).^1^ Following the global eradication of wild-type 2 poliovirus, the risk of vaccine-associated paralytic polio and cVDPVs led to the global withdrawal of type 2 virus from OPVs for routine use in April, 2016, with a switch to use of bivalent OPVs containing only types 1 and 3.^2^ Since this switch routine immunisation against type 2 poliovirus has been exclusively through the use of trivalent inactivated poliovirus vaccines (IPVs).

However, despite the switch and cessation of routine use of live type 2 OPVs (OPV2), type 2 cVDPV (cVDPV2) outbreaks have occurred in many countries, resulting in cases of acute flaccid paralysis, which represent the majority of such polio cases worldwide.^3^ Because IPVs alone, the only existing source of protection against type 2 poliovirus in routine immunisation, induce minimal intestinal immunity, these vaccines are ineffective in preventing faecal–oral transmission in settings of poor hygiene and sanitation.^4^ The only way to contain cVDPV2 outbreaks in such settings is to use stockpiled monovalent OPV2 vaccine, which itself risks propagating new cVDPVs. A novel OPV2 (nOPV2) is an important new addition to outbreak control tools. nOPV2 has been genetically designed with attenuating modifications and shown to have a lower risk of reversion to neurovirulence compared with the Sabin vaccine, but with non-inferior immunogenicity in adults and children.^5^, ^6^, ^7^

**Research in context**

**Evidence before this study**

Outbreaks of circulating vaccine-derived poliovirus type 2 (cVDPV2) are now the main cause of paralytic poliomyelitis worldwide. A novel, genetically more stable monovalent type 2 oral polio vaccine that was developed for use in outbreak control with less inherent chance of propagating more cVDPV2 has been studied in clinical trials involving infants, children, adolescents, and adults previously primed with at least one dose of type 2-containing poliovirus vaccine and shown to be safe, well tolerated, and immunogenic. On the basis of this clinical evidence, WHO authorised the use of a novel oral poliovirus vaccine type 2 (nOPV2) for outbreak control through the emergency use listing (EUL) procedure and more than 450 million doses have been distributed for use since March 2021. However, no clinical trial data are available for the use of nOPV2 in poliovirus vaccine-naive infants, who are at particular risk of infection during cVDPV2 outbreaks.

**Added value of this study**

The EUL-enabling clinical data on nOPV2 included safety and immunogenicity data in infants who had already received a dose of inactivated poliovirus vaccine. Thus, generating vaccine performance data in poliovirus vaccine-naive infants or newborn babies was important to complete the clinical database on the new vaccine. Moreover, the highest-risk groups in cVDPV outbreaks are those with incomplete or no poliovirus vaccination such as newborn and very young infants. These groups will be priority targets in vaccination campaigns with nOPV2 for cVDPV2 outbreak control. Our data showing that nOPV2 is safe, well tolerated, and immunogenic in newborn infants are important for those health-care administrators and decision makers responsible for implementing such campaigns as well as being reassuring for the parents and carers of affected children. Equally important is our observation that nOPV2 use in newborn infants will not lead to excessive faecal excretion of live nOPV2, further minimising the risks of propagating a cVDPV2 outbreak.

**Implications of all the available evidence**

Since WHO declared that cVDPV2 outbreaks are a public health emergency of international concern and following submission of earlier clinical data on nOPV2 in individuals aged at least 18 weeks to adults, WHO authorised the distribution and use of the vaccine in such outbreaks as the first vaccine to be used under the EUL process. To date, more than 450 million doses of nOPV2 have been distributed and have been used in all age groups targeted for outbreak response with no exclusion of use in newborn infants. The data from this study can inform policy makers, regulators, and health-care providers that the use of nOPV2 in poliovirus vaccine-naive newborn infants, the age group considered most vulnerable for poliovirus transmission, is safe and immunogenic.

The clinical development of nOPV2 is ongoing, but the successful demonstration of its safety, immunogenicity, and genetic stability in phase 1 and phase 2 studies coupled with the public health emergency of international concern constituted by cVDPV2 outbreaks led to the authorisation by WHO of the vaccine's use under WHO's emergency use listing (EUL) procedure. To date, over 450 million doses have been distributed for outbreak control.^8^ Clinical studies showing the safety, tolerability, and immunogenicity of nOPV2 were all done in adults and children previously vaccinated against polio.^5^, ^6^, ^7^ The investigation of virus obtained in stool samples from these studies showed superior genetic stability, with nOPV2 less likely to revert to a neurovirulent phenotype, relative to Sabin-strain OPV2. Because no age restriction exists for the use of nOPV2 in field conditions of outbreak control, many of the at-risk population will be vaccine-naive newborn infants, a subpopulation in which no clinical data with nOPV2 exist. IPV-only populations were previously used as surrogates for vaccine-naive infants to allow early use of nOPV2 in newborn infants. For this reason, the EUL required assessments of the safety, tolerability, and immunogenicity of the vaccine in vaccine-naive newborn infants. We aimed to provide this crucial information and to inform policy regarding nOPV2 use, including an assessment of the shedding of the vaccine virus.

## Methods

### Study design and participants

We did a randomised, double-blind, phase 2 clinical study at the Matlab Health Research Center in Chandpur, rural Bangladesh. The protocol was approved by the research review committee and the ethical review committee at the International Centre for Diarrhoeal Disease Research, Bangladesh (icddr,b).

Women in the third trimester of their pregnancy were identified by local community health workers and had study objectives and procedures explained to them before they were invited to enrol their infant. Enlisted pregnant women were requested to give birth at the Matlab hospital or subcentre, icddrb, and were asked to enrol their newborn infant for study vaccination within 0–3 days of birth. Eligibility criteria for the mother required willingness and ability to comply with the study procedures and provision of consent for their infant to participate for the entire duration of the study. Eligibility criteria for newborn infants at screening were that they were healthy and were a single birth after at least 37 weeks' gestation. Major exclusion criteria included receipt of rotavirus or any other poliovirus vaccine, any infection or illness at the time of enrolment (vomiting, diarrhoea, or intolerance to liquids), diagnosis or suspicion of any immunodeficiency disorder in the infant or a close family member, or any contraindication for venipuncture (appendix p 3). The study schedule necessitated a 2-week delay in polio vaccination through the local Expanded Programme on Immunization (EPI; normally 6, 10, and 14 weeks) but, in the absence of circulating polioviruses (the WHO South-East Asia region was certified as polio-free in March 2014^9^), this delay was not considered to pose a risk to participants.

This study was done in compliance with the relevant regulatory requirements and the International Council for Harmonisation Good Clinical Practice guidelines of the Declaration of Helsinki for biomedical research involving human subjects under the guidance of the icddr,b data and safety monitoring board. All parents and guardians who volunteered their children to participate provided written informed consent before enrolment. This study is registered on ClinicalTrials.gov, NCT04693286.

### Randomisation and masking

Participants were randomly assigned (2:1) to either nOPV2 or placebo, using a block randomisation of size 6 or 12 generated by a statistician who was not otherwise involved in the study. The statistician prepared randomisation lists for study nurses or investigators who were masked to the allocation of the vaccine or placebo. Parents, investigators, and laboratory personnel responsible for the various analyses remained masked to group allocation until the end of the study.

### Procedures

Infants in the nOPV2 group received a vaccine (PT Bio Farma, Indonesia) composed of an attenuated serotype 2 poliovirus derived from a modified Sabin 2 infectious clone propagated in Vero cells.^10^ The lot used contained 10^5·0±0·5^ cell culture infectious dose of 50% (CCID_50_) of the genetically modified type 2 poliovirus in each dose, a dose representing minimum potency at expiration, administered as two drops (0·1 mL) in the mouth using a supplied dropper. Infants in the placebo group received sucrose in Basal Medium Eagle media and buffer (Bio Farma, Indonesia). EPI vaccines recommended by the Bangladesh schedule were administered at age 8, 12 and 16 weeks (appendix p 2).

The first vaccine or placebo dose was given within a window of 0–3 days after birth and all participants received a second dose of their assigned vaccine or placebo 4 weeks after the first dose. Because this was the first planned use in newborn infants, study administrations were first given to 30 participants in a blinded manner (20 [67%] participants with nOPV2 and ten [33%] participants with placebo) and the safety data from these participants were reviewed by the data and safety monitoring board before approval to continue vaccination of the remaining infants.

Following vaccine or placebo administration participants were observed for 30 min to record any immediate reaction or adverse event. After each vaccination study staff visited participants' homes daily for 7 days to record in electronic diaries solicited systemic adverse events. Mothers of participants were provided with a telephone number to communicate with the investigator or study staff to report any serious adverse events and weekly study visits were continued throughout the study duration to collect and record any reports of unsolicited adverse events, serious adverse events, or adverse events of special interest, notably anaphylactic reactions, aseptic meningitis or encephalitis, unexplained deaths, or acute flaccid paralysis due to cVDPV or vaccine-associated paralytic polio. The intensity of any recorded solicited or unsolicited adverse event was assessed by the investigator using a standard grading scheme (appendix p 3).

Before the vaccine administrations at birth and 4-week visits, 1-mL blood samples were drawn and kept refrigerated (2–8°C) during shipping to the icddr,b Matlab laboratory, where sera were prepared within 24 h. Serum aliquots were stored at –20°C and shipped to the Polio and Picornavirus Laboratory of the Division of Viral Diseases, Centers for Disease Control and Prevention (CDC), Atlanta, GA, USA for measurement of neutralising antibodies against the three polio types by microneutralisation assay. Antibody titres below 1:8 were considered non-detectable and the highest reported reciprocal titre was calculated as at least 1:1448 (10·5 log_2_).^11^

Mothers were asked to collect samples of approximately 8 g of stool at birth and at 2, 4, 6, 8, 10, and 12 weeks during the study (appendix p 2). Stool samples were kept refrigerated (2–8°C) during shipping to the icddr,b Matlab laboratory, where aliquots were prepared and stored at –20°C and shipped to the CDC laboratory to qualitatively detect polioviruses by real-time RT-PCR. Quantitative measurements of infectious virus were done in samples positive by RT-PCR and expressed as CCID_50_ per g of stool.^12^

### Outcomes

The coprimary objectives were to assess the safety and tolerability, and the immunogenicity (measured by seroconversion rate) after one and two doses of nOPV2 when given 4 weeks apart in poliovirus vaccine-naive newborn infants. The secondary outcome was an assessment of the rate, duration, and extent of faecal viral shedding measured by real-time RT-PCR.

The safety and tolerability objective was assessed as the incidence rates of solicited adverse events in the 7 days after each vaccination, and unsolicited adverse events, serious adverse events, and adverse events of special interest over the entire study duration, with comparison between nOPV2 and placebo groups. The primary immunogenicity outcome was the seroconversion rate for neutralising antibodies against type 2 poliovirus measured 28 days after the first and second vaccinations with nOPV2. We defined the seroconversion rate cumulatively as the proportions for each group who either (1) became seropositive (≥1:8 titre) after being seronegative (<1:8) at baseline, or (2) had a four-fold or greater increase above the predicted titre in baseline seropositive participants. The predicted titre was calculated assuming an exponential decay from baseline with a half-life of 28 days.

### Statistical analysis

We selected a sample size of 220 participants in the nOPV2 group, with a placebo control group with half that number of participants (n=110), to provide sufficient power to assess the immunogenicity of the candidate vaccines on the basis of previous studies. In a study done in India in 2008, before the cessation of OPV2 usage, Sutter and colleagues^13^ found that two doses of monovalent OPV2 administered at birth and 30 days later had a cumulative seroconversion rate of 90% after the second dose. For the present study we conservatively assumed that the seroconversion rate for nOPV2 would be 85% versus the 90% in the study from India, the decrement owing to lack of passive exposure to OPV2. The sample size for the nOPV2 group was selected so that an observed seroconversion rate of 85% would have a two-sided score-based 95% CI width of 10% (plus or minus 5%). We required 196 evaluable participants in the nOPV2 group, which was increased to 220 to allow for a dropout rate of approximately 10%. Simulations indicated that with this sample size, if the true seroconversion rate was decreased by 10% or more (up to ≤80%) from that of Sutter and colleagues' study,^13^ the upper bound of the 95% CI would exclude a seroconversion rate of 90% with greater than 98% probability, which was considered a sufficient level of precision to assess the immunogenicity of nOPV2. With 220 participants in the nOPV2 group assumed to contribute to the safety assessment, the study had sensitivity to detect adverse events in 0·5% of participants with 67% probability, in 1·0% with 89% probability, and 2·0% with 99% probability. The secondary objective viral shedding is presented descriptively as the magnitude and duration of viral shedding as identified by real-time RT-PCR. Safety analyses are presented based on the intention-to-treat population (all participants who received at least one study intervention), and immunogenicity analyses on the per protocol population (all those who received both study interventions and had no serious protocol violation. We estimated geometric mean titres with likelihood-based methods, incorporating left (right) censoring at assay lower limit of quantitation (2·5 log_2_) or upper limit of quantitation (ULOQ; 10·5 log_2_), assuming normal error on the log_2_ scale. All statistical analyses were done using SAS (versions 9.3 and 9.4).

### Role of the funding source

Four authors, KZ, JDC, AGR, and MH, were employees of the study sponsor, and one, ASB, is employed by the study funder, who were involved in study design, data collection, and writing of the study report.

## Results

**Figure 1:**
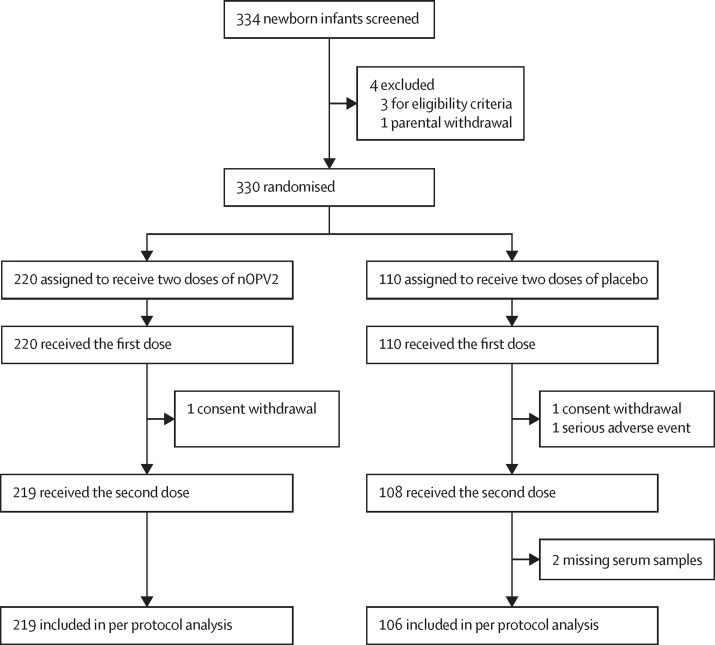
Trial profile nOPV2=novel oral poliovirus vaccine type 2.

Between Sept 21, 2020, and Aug 16, 2021, we screened 334 newborn infants, of whom three (<1%) were found to be ineligible and one (<1%) was withdrawn by the parents; the remaining 330 (99%) infants were assigned to the two study groups (220 [67%] assigned to nOPV2 and 110 [33%] to placebo; figure 1). Demographics of the two study groups were similar with regard to sex, weight, and length of the newborn infants (table 1). Similar proportions of the two study groups received BCG vaccine at birth. We had good compliance with completion of the study, with 327 (99%) of 330 participants receiving both doses of vaccine or placebo and 325 (98%) available for the per protocol analysis of immunogenicity (figure 1).

**Table 1:** Demographics of study participants at enrolment (intention-to-treat population)

		**nOPV2 (n=220)**	**Placebo (n=110)**
Age, days		1·1 (0·7)	1·1 (0·7)
Sex			
	Male	99 (45%)	56 (51%)
	Female	121 (55%)	54 (49%)
Weight, kg		2·9 (0·3)	2·9 (0·4)
Length, cm		48·3 (1·6)	48·5 (1·9)
Breastfeeding			
	At enrolment	219/200 (>99%)	110/110 (100%)
	At week 8	219/219 (100%)	107/107 (100%)
Received BCG vaccine			
	At birth (visit 1)	87 (40%)	49 (45%)
Received any other vaccine*			
	At birth (visit 1)	1 (<1%)	0
	At age 4 weeks (visit 3)	50 (23%)	22 (20%)
	At age 8 weeks (visit 5)	2 (1%)	1 (1%)

Data are n (%), n/N (%), or mean (SD). nOPV2=novel oral poliovirus vaccine type 2.

* Other vaccines included one case of a prophylactic dose of hepatitis A/B at birth.

**Figure 2:**
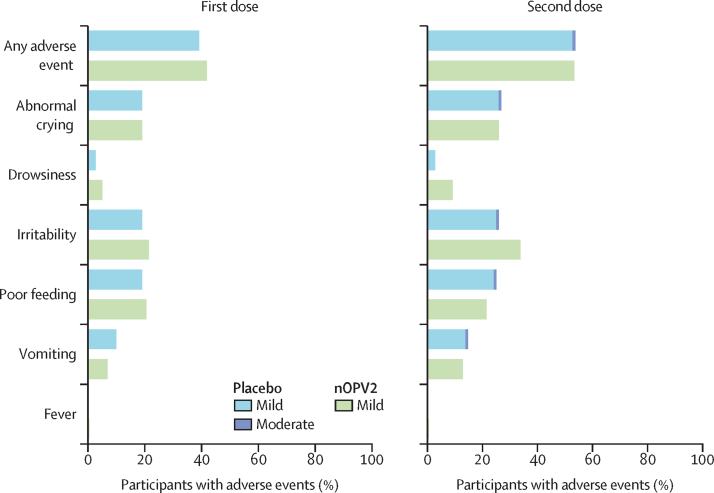
Rates of solicited adverse events within 7 days of the first and second doses in the nOPV2 and placebo groups Severity shown only for placebo group as all adverse events in the nOPV2 group were reported as mild. nOPV2=novel oral poliovirus vaccine type 2.

We found that nOPV2 was as well tolerated as placebo; adverse events were reported in 183 (83%) of 220 nOPV2 recipients and in 90 (82%) of 110 placebo recipients (appendix p 4). We found no immediate reactions within 30 min of vaccination and solicited adverse events that were reported in 154 (70%) nOPV2 recipients and 78 (71%) placebo recipients were all mild or moderate, with no severe cases reported (figure 2). After the first dose incidence rates in the two study groups were similar and all events were mild in intensity (figure 2). Rates increased slightly after the second dose but were exclusively mild in the nOPV2 group with a few events in one participant described as moderate in the placebo group (figure 2). The most frequent solicited events after each dose of nOPV2 or placebo were irritability, abnormal crying, and poor feeding (figure 2). Unsolicited adverse events were reported in 87 (40%) infants in the nOPV2 group and in 38 (35%) in the placebo group (appendix p 4). Severe unsolicited adverse events were reported in 11 (5%) nOPV2 recipients and in five (5%) placebo recipients, mostly comprising respiratory disorders (appendix p 4). The most frequent of these was pneumonia, in seven nOPV2 recipients and four placebo recipients (appendix p 5). Other severe events in the nOPV2 group were two cases of bronchiolitis, one respiratory tract infection, and one case of pyrexia; one case of aspiration pneumonia that was considered severe occurred in the placebo group (appendix p 5). No severe unsolicited adverse events were considered to be related to study treatment.

Most of the severe unsolicited adverse events were also considered to be serious adverse events. In 16 participants we reported 19 serious adverse events, none of which were considered to be related to study treatment when assessed by the study investigators. With the exception of one infant in the nOPV2 group who was diagnosed with severe pneumonia 5 days after the second vaccination, all serious adverse events occurred more than 9 days after receiving a first or second dose of nOPV2 or placebo. We reported no adverse events of special interest, but when unsolicited adverse events were specifically analysed for gastrointestinal disorders that could potentially be related to an oral vaccine we found six adverse events in nOPV2 recipients: five mild cases (one of vomiting, two of abdominal distension, and two of watery diarrhoea) and one umbilical hernia described as moderate in severity. In placebo recipients we found one case of mild watery diarrhoea. Investigators did not consider that any of these gastrointestinal disorders were related to vaccination.

**Figure 3:**
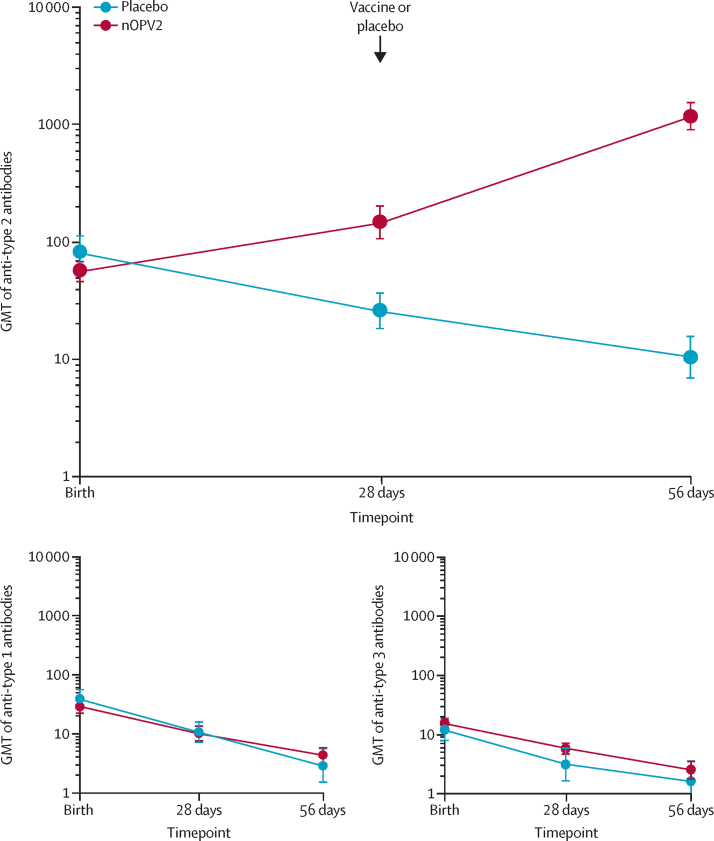
Neutralising antibody responses to two doses of nOPV2 or placebo Large panel shows GMTs against poliovirus type 2 and smaller panels show GMTs against poliovirus types 1 and 3 (appendix p 6). Error bars denote 95% CIs. GMT=geometric mean titre. nOPV2=novel oral poliovirus vaccine type 2.

Most newborn infants had neutralising antibodies against polioviruses at birth, presumed due to transfer of maternal antibodies. Seroprotective titres (≥1:8) were present in 204 (93%) of 220 infants in the nOPV2 group and 102 (93%) of 110 in the placebo group (table 2). Waning of maternal antibodies was evident in the placebo group, with 81 (75%) of 108 having protective titres at week 4 and 55 (56%) of 104 at week 8, with a corresponding decrease in titres (figure 3; appendix p 6). Against this background, nOPV2 elicited seroconversion in 100 (46%) of 219 vaccine recipients after one dose, and in 196 (90%) of 217 after two doses (table 2), with an increase in titres (figure 3). When corrected for the possibility of accurately observing seroconversion, because four times the predicted maternal antibody titre would be less than the assay ULOQ, the result was similar, with seroconversion in 100 (48%) of 210 participants 4 weeks after the first dose. The result was the same at 8 weeks as predicted titres for all participants then fell below the ULOQ.

**Table 2:** Seroconversion and seroprotection rates for type 2 poliovirus neutralising antibodies (per protocol)

	**nOPV2**	**Placebo**
**Seroconversion rates**		
Type 2 at 4 weeks	100/219 (45·7%; 38·9–52·5)	1/108 (0·9%; 0·0–5·1)
Type 2 at 8 weeks	196/217 (90·3%; 85·6–93·9)	2/104 (1·9%; 0·2–6·8)
Type 2 at 4 weeks in those with low enough baseline titres*	100/210 (47·6%; 40·7–54·6)	1/99 (1·0%; 0·0–5·5)
**Seroprotection rates**		
Type 2 at birth	204/220 (92·7%; 88·5–95·8)	102/110 (92·7%; 86·2–96·8)
Type 2 at 4 weeks	198/219 (90·4%; 85·7–94·0)	81/108 (75·0%; 65·7–82·8)
Type 2 at 8 weeks	214/217 (98·6%; 96·0–99·7)	58/104 (55·8%; 45·7–65·5)

Data are n/N (%; 95% CI). nOPV2=novel oral poliovirus vaccine type 2.

* Only measured in participants whose baseline titre was low enough to allow measurement of a four-times increase without exceeding the maximum titre of 1448. All participants qualified at week 8.

Seroconversion resulted in 214 (99%) of 217 vaccine recipients having seroprotective titres (≥1:8) at 8 weeks, after this proportion had initially reduced to 198 (90%) of 219 vaccine recipients at week 4 (table 2). Of the 16 infants in the nOPV2 group who were initially unprotected at birth with no detectable titres, eight (50%) had seroprotective titres at week 4 and all 16 (100%) infants reached this level by week 8 (table 2). In the placebo group, one (1%) of 108 participants seroconverted after 4 weeks, and two (2%) of 104 seroconverted after 8 weeks (table 2). One participant had a baseline titre of 588 which increased to 1176 at week 4, which might have been a true seroconversion but was only one dilution step difference (table 2). However, the other two observations were probably artifactual because both had baseline titres greater than the ULOQ and still had these titres at week 8 when predicted maternal titres would be 362 based on a four-times decrease, so were counted as seroconverted according to the definition.

Titres of neutralising antibodies against poliovirus types 1 and 3 decreased from birth to week 8 in both nOPV2 and placebo groups, with no notable differences between groups (figure 3); respective seroprotection rates for type 1 were 75% (164/220) in the nOPV2 group and 77% (85/110) in the placebo group at birth, which decreased to 37% (81/217) in the nOPV2 group and 34% (36/106) in the placebo group by week 8. For type 3 poliovirus, corresponding seroprotection rates decreased from 66% (145/220) in the nOPV2 group and 57% (63/110) in the placebo group at birth to 18% (40/217) in the nOPV2 group and 25% (27/106) in the placebo group by week 8. Geometric mean titres are shown in the appendix (p 6). The small differences in numbers between intention-to-treat and per protocol populations mean that immunogenicity analyses were not different between the two populations (data not shown).

With respect to the secondary objective of viral shedding, as expected, we found no RT-PCR-detectable shedding of poliovirus types 1, 2, or 3 in baseline stool samples; however, 2 weeks after the first dose of nOPV2, type 2 viral shedding was detectable in 114 (52%) of 219 nOPV2 recipients (table 3). After decreasing to 40% (87/219) at week 4, detectable viral shedding increased again to 64% (140/219) at week 6 following administration of the second dose of nOPV2, before gradually decreasing to week 12 when only one participant was still shedding detectable virus (table 3). Across all study visits, we found type 2 viral shedding in three placebo recipients, one at age 2 weeks and two at 6 weeks. Notably, these were not the same infants who were found to have seroconverted and this finding might be due to cross-contamination of samples, or less plausibly passive exposure to nOPV2 from vaccinees living in the same neighbourhood.

**Table 3:** Rates of faecal viral shedding by type assessed by real-time RT-PCR over the course of the study (per protocol)

	**Type 2 PCR positive**		**Type 2 PCR positive or CCID**_50_**/g**		**Type 1 PCR positive**		**Type 3 PCR positive**	
	nOPV2 (n=219)	Placebo (n=110)*	nOPV2 (n=219)	Placebo (n=110)*	nOPV2 (n=219)	Placebo (n=110)*	nOPV2 (n=219)	Placebo (n=110)*
**nOPV2 or placebo**								
Baseline (at birth)	0 (0·0%; 0·0–1·7)	0 (0·0%; 0·0–3·3)	0 (0·0%; 0·0–1·7)	0 (0·0%; 0·0–3·3)	0 (0·0%; 0·0–1·7)	0 (0·0%; 0·0–3·3)	0 (0·0%; 0·0–1·7)	0 (0·0%; 0·0–3·3)
2 weeks	114 (52·1%; 45·2–58·8)	1 (0·9%; 0·0–3·3)	38 (17·4%; 12·6–23·0)	0 (0·0%; 0·0–3·3)	0 (0·0%; 0·0–1·7)	0 (0·0%; 0·0–3·3)	0 (0·0%; 0·0–1·7)	0 (0·0%; 0·0–3·3)
4 weeks	87 (39·7%; 33·2–46·5)	0 (0·0%; 0·0–3·43)	2 (0·9%; 0·1–3·3)	0 (0·0%; 0·0–3·4)	0 (0·0%; 0·0–1·7)	0 (0·0%; 0·0–3·4)	0 (0·0%; 0·0–1·7)	0 (0·0%; 0·0–3·4)
6 weeks	140 (63·9%; 57·2–70·3)	2 (1·9%; 0·2–6·5)	30 (13·7%; 9·4–19·0)	0 (0·0%; 0·0–3·4)	0 (0·0%; 0·0–1·7)	1 (0·9%; 0·0–5·1)	0 (0·0%; 0·0–1·7)	0 (0·0%; 0·0–3·4)
**Bivalent OPV plus** fractional IPV								
8 weeks	84 (38·4; [31·9–45·1)	0 (0·0%; 0·0–3·5)	5 (2·3; 0·.7–5·2)	0 (0·0; 0·0–3·5)	0 (0·0%; 0·0–1·7)	1 (1·0%; 0·0–5·2)	2 (0·9%; 0·1–3·3)	0 (0·0%; 0·0–3·5)
10 weeks	5 (2·3%; 0·7–5·2)	0 (0·0%; 0·0–3·4)	0 (0·0%; 0·0–1·7)	0 (0·0%; 0·0–3·4)	124 (56·6%; 49·8–63·3)	77 (72·0%; 62·5–80·2)	146 (66·7%; 60·0–72·9)	71 (66·4%; 56·6–75·2)
12 weeks	1 (0·5%; 0·0–2·5)	0 (0·0%; 0·0–3·4)	0 (0; 0·0–1·7)	0 (0·0%; 0·0–3·4)	60 (27·4%; 21·6–33·8)	43 (40·2%; 30·8–50·1)	122 (55·7%; 48·9–62·4)	60 (56·1%; 46·1–65·7)

CCID50=cell culture infectious dose of 50%. IPV=inactivated poliovirus vaccine. nOPV2=novel oral poliovirus vaccine type 2. OPV=oral poliovirus vaccine.

* n=110 at baseline and week 2, n=108 at weeks 4 and 6, n=105 at week 8, and n=107 at weeks 10 and 12.

The peak positivity of 17% (38/219) in the CCID_50_ culture assay 2 weeks after the first nOPV2 dose (table 3) indicates lower quantities of virus shed relative to those shed by older infants in the phase 2 study.^14^ The rate decreased to 14% (30/219) 2 weeks after the second dose of nOPV2 and to zero by 10 weeks and we found no such shedding in the placebo group. Two infants in the nOPV2 group had stool samples that contained at least 4·0 log_10_ CCID_50_/g, one at weeks 2, 4, and 6, and another at week 6.

We found two instances each of shedding of poliovirus types 1 and 3 before the administration of bivalent OPV at week 8, with stool samples from two different placebo recipients being PCR positive for type 1 at weeks 6 and 8, and two nOPV2 vaccinees with samples PCR positive for type 3 at week 8. Following doses of bivalent OPV and fractional IPV administration at week 8, poliovirus types 1 and 3 were detected by PCR, but with a small difference in the type 1 shedding in nOPV2 and placebo recipients. 2 weeks after bivalent OPV administration, 77 (72%) of 107 infants in the placebo group were shedding PCR-detectable type 1 poliovirus, which decreased to 43 (40%) of 107 by 12 weeks. In the nOPV2 group, these rates were lower, at 57% (124/219) at week 10 and 27% (60/219) at week 12. Shedding of type 3 poliovirus was similar in both groups and appeared to last longer than that of type 1. Thus, type 3 poliovirus was detectable in 146 (67%) of 219 infants in the nOPV2 group and 71 (66%) of 107 in the placebo group at week 10 and was still detectable in 122 (56%) infants in the nOPV2 group and 60 (56%) in the placebo group at week 12.

## Discussion

We found similar tolerability profiles in the nOPV2 and placebo groups, with only mild solicited adverse events and no related serious adverse events or adverse events of special interest reported. Solicited adverse event rates after nOPV2 and placebo were higher after second doses than after first doses, representing either an increase in the detection rate of such events in children aged 4–5 weeks compared with 0–10 days, or a true increase in the event rate. The immune response was shown against a background of waning maternal neutralising antibodies, evidenced in the placebo group, in which the seroprotection rate decreased from 93% at baseline to 56% at week 8. The 90% seroconversion rate elicited by two doses of nOPV2 resulted in 99% of nOPV2 recipients having seroprotective titres at week 8. Furthermore, in those infants who did not have protective maternal antibodies at birth, vaccination with nOPV2 resulted in 100% seroconversion after two doses, indicating that all infants had protective titres.

Following the global withdrawal of OPV2 vaccines,^2^ we could not include a Sabin-strain monovalent OPV2 control group in this study. The observed immune responses correspond well with those observed in newborn infants in India by Sutter and colleagues.^13^ Of 830 Indian infants, 703 (85%) had seroprotective titres against type 2 at birth, and seroconversion rates were 21% after one dose and 90% after two doses of monovalent OPV2, with generally higher neutralising antibody concentrations following nOPV2. Importantly, nOPV2 had a target dose of 10^5·0 ±0.5^ CCID_50_, whereas the Sabin monovalent OPV2 used by Sutter and colleagues contained poliovirus type 2 of at least 10^5^ CCID_50_ at release. Direct comparisons with the Indian study cannot be made because of multiple confounding factors, but it is encouraging that immune responses appear similar or higher after one or two doses of this low-dose nOPV2, which was previously immunobridged to monovalent OPV2 in the phase 2 study.^7^ Seroconversion was rare in the placebo group, consistent with anticipation that seroconversion would only be observed in rare cases owing to random assay variability in a setting such as Bangladesh that does not have any ongoing use or known circulation of poliovirus type 2. In all other respects, including the tolerability and safety and the immune responses to poliovirus types 1 and 3, our results with nOPV2 in Bangladeshi newborn infants correspond well with those reported in Indian newborn infants with monovalent OPV2.^4^, ^13^

Proportions of infants shedding type 2 virus 2 weeks after the first and second doses of nOPV2 were similar to the proportions shedding types 1 and 3 viruses 2 weeks after the first dose of bivalent OPV. These proportions declined progressively through weeks 4, 8, and 10, and only one infant was still shedding type 2 virus by week 12.

Amounts of virus excreted were low, with only 17% at week 2 and 14% at week 6 shedding sufficient virus to be measurable by culture, 2 weeks after the nOPV2 doses, indicating no increased transmission risk for those receiving nOPV2 before any other poliovirus vaccination, relative to those previously receiving IPV.^14^ These proportions had declined rapidly by 4 weeks after each dose. This finding is in contrast with the viral shedding data from the Indian study,^13^ in which a very low rate of shedding, measured by culture, was found 7 days after a first monovalent OPV2 dose (<10%) and peak shedding occurred 7 days after the second dose (approximately 75%). The two studies differ in several aspects: shedding was measured by different laboratories and Sutter and colleagues did not include PCR-based shedding assessment, Sutter and colleagues vaccinated at birth whereas we had a window of the first 3 days after birth to vaccinate, and perhaps most importantly Sutter and colleagues measured shedding 1 week after vaccination whereas we collected stool samples 2 and 4 weeks after each vaccination. In our later phase 2 studies we found that culture-positive shedding was higher at 7 days than at 14 days after the first dose.^14^

However, our data correspond well with previous observations of nOPV2 shedding in the phase 2 study in previously vaccinated infants in which infants who received nOPV2 displayed similar shedding characteristics to those who received monovalent OPV2 7 days after vaccination but then a significantly lower rate of shedding than monovalent OPV2 4 weeks after vaccination.^7^, ^14^ Viral shedding of nOPV2 in this population was low compared with in the Indian study, although several differences existed in the study design, epidemiological background, and study settings. This finding could be an indication of lower risk of sustained transmission and, ultimately, lower risk of emergence of cVDPV when using nOPV2 compared with monovalent OPV2. Also, the overall pattern of shedding with an initial peak within 2 weeks decreasing to lower levels by 4 weeks was consistent with the pattern observed over decades with OPVs in various clinical studies.^4^ Notably, shedding of type 1 virus after the first dose of bivalent OPV was lower in the nOPV2 group than in the placebo group, whereas shedding of type 3 virus was similar in both groups. Sabin type 2 virus interferes with both viral replication and humoral responses of types 1 and 3, with some more limited interference between type 1 and type 3.^13^, ^15^, ^16^, ^17^ Reduction of interference was an additional advantage when type 2 virus was withdrawn from trivalent OPV and substituted with bivalent OPV.^2^

We have no data on whether nOPV2 interferes with the viral replication or immune responses to Sabin types 1 and 3, as would occur with the later administration of bivalent OPV in routine vaccination schedules. This gap could be seen as a limitation of the present study, because we did not investigate the immune responses to bivalent OPV or the fractional dose IPV, but this question is being investigated in a parallel study (NCT04579510). An additional limitation common to this and other clinical trials of vaccines is that generalisability to subpopulations with comorbidities is generally difficult, particularly those with immune deficiencies.

Concerns in industrialised nations where poliovirus is considered to be eliminated following the detection of cVDPV2 in sewage in London, UK,^18^ and in Israel^19^ have been amplified by the occurrence of a case of paralytic poliomyelitis due to cVDPV2 in an unvaccinated adult in Rockland County, NY, USA, in a local setting with low poliovirus vaccination coverage.^20^ These reports show that cVDPV is an international concern, not restricted only to countries with low immunisation coverage, and there are concerns that transmission might be widespread.^21^ nOPV2 might be an option for outbreak control in such situations.^8^ Extensive review of the use of Sabin OPV and Salk IPV vaccines in the first 7 days of life has not previously revealed any safety concerns,^22^ and it is reassuring to observe that there are no safety signals evident from the prospective use of nOPV2 in newborn infants in the present clinical study.

Data from this study support the use of nOPV2 in vulnerable vaccine-naive newborn infants, who might be at particular risk during cVDPV2 outbreaks. nOPV2 has already been widely implemented in outbreak responses, with over 450 million doses distributed under the WHO EUL procedure, with no restriction on the age of recipients.^8^ The data from this study support the continued use of nOPV2 in outbreak response for a designated global health emergency.

## Data sharing

Data for this study will be made available to others in the scientific community upon request. Standard criteria for making data available for valid research projects will be used, following application by suitably qualified researchers. For data access, please contact the Bill & Melinda Gates Foundationat openaccess@gatesfoundation.org.

## Declaration of interests

ET is a full-time employee of the vaccine manufacturer, PT Bio Farma (Bandung, Indonesia). All other authors declare no competing interests.
