# Supplementary material for: Evaluation of the safety, immunogenicity, and faecal shedding of novel oral polio vaccine type 2 in healthy newborn infants in Bangladesh: a randomised, controlled, phase 2 clinical trial
Source: Lancet. 2023 Jan 14;401(10371):131–9. doi: 10.1016/S0140-6736(22)02397-2 (PMC9860215; doi:10.1016/S0140-6736(22)02397-2)
Supplement: Supplementary appendix [file mmc1.pdf]

THE LANCET

Supplementary appendix

This appendix formed part of the original submission and has been peer reviewed. We post it as supplied by the authors.

Supplement to: Zaman K, Bandyopadhyay AS, Hoque M, et al. Evaluation of the safety, immunogenicity, and faecal shedding of novel oral polio vaccine type 2 in healthy newborn infants in Bangladesh: a randomised, controlled, phase 2 clinical trial. *Lancet* 2022; published online Dec 7. https://doi.org/10.1016/S0140-6736(22)02397-2 .

Supplementary appendix

Safety, immunogenicity, and faecal shedding of novel oral poliovirus vaccine type 2 (nOPV2) in healthy newborn infants in Bangladesh: a randomised, controlled, phase 2, clinical trial

K. Zaman et al

**Supplementary figure 1.**	Schematic design of the study	page 2
**Inclusion/exclusion criteria**		page 3
**Supplementary table 1.**	Intensity grading of solicited and unsolicited AEs	page 4
**Supplementary table 2.**	Adverse events reported during the study	page 5
**Supplementary table 3.**	Unsolicited adverse events	page 6
**Supplementary table 4.**	GMTs of polio neutralising antibodies	page 7

Supplementary Figure 1. Schematic design of the study

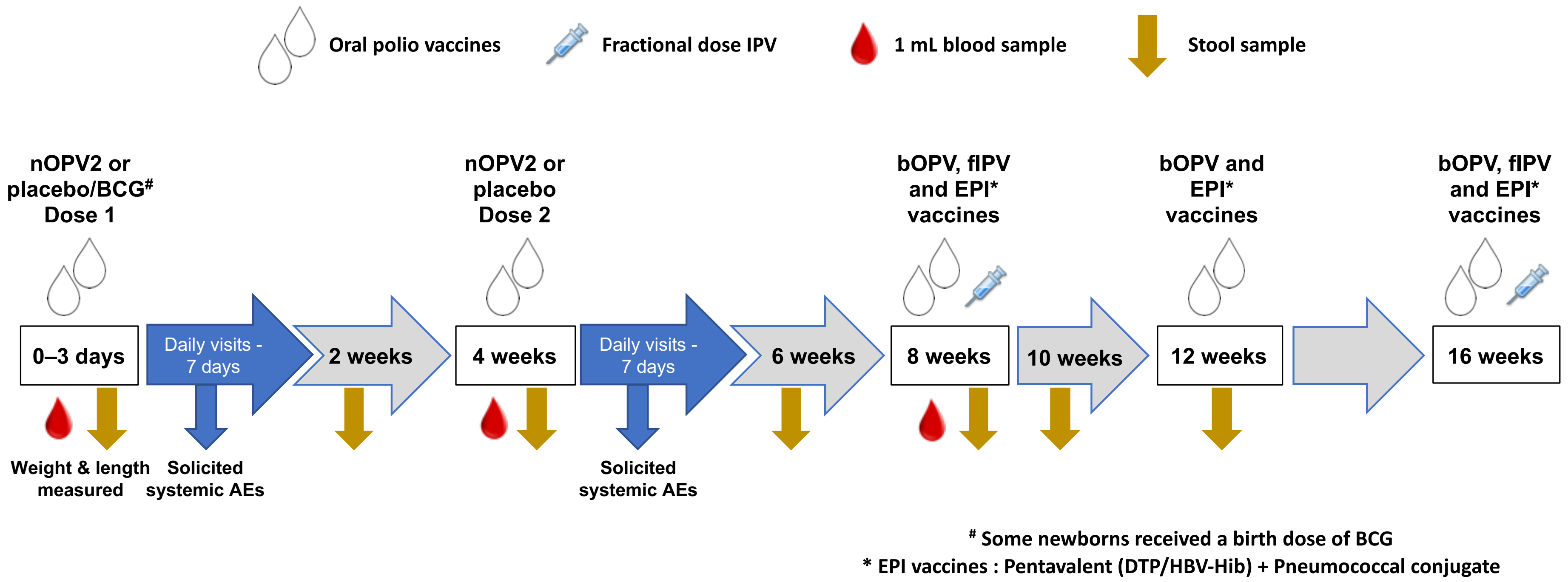

Inclusion and exclusion criteria

Inclusion Criteria

Participants who met the following criteria were considered eligible to participate in the clinical study:

1. Newborns at birth (range: 0–3 days of age)
2. Mothers that consented for participation in the full length of the study
3. Mothers that were able to understand and comply with planned study procedures

Exclusion Criteria

Participants who met one or more of the following criteria were not considered eligible to participate in the clinical study:

1. Mothers and newborns who were unable to participate in the full length of the study
2. A diagnosis or suspicion of immunodeficiency disorder either in the newborn or in an immediate family member
3. A diagnosis or suspicion of a bleeding disorder that would contraindicate collection of blood by venipuncture
4. Acute diarrhoea, infection or illness at the time of enrolment that would require admission to a hospital
5. Acute vomiting and intolerance to liquids within 24 hours before the enrolment visit
6. Receipt of any polio vaccine (OPV or IPV) and rotavirus vaccine before enrolment based upon documentation or mothers' recall
7. Newborns from multiple births. Newborns from multiple births were excluded to reduce the potential for contact transmission of vaccine poliovirus to siblings
8. Newborns from premature births (<37 weeks of gestation)

Supplementary table 1: Intensity scales for solicited and unsolicited adverse events

Events	Severity Grade			
	**0 (None)**	**1 (Mild)**	**2 (Moderate)**	**3 (Severe)**
**Solicited Adverse Events**				
**Fever**	< 37·5°C	37·5°–38·0°C	38·1°–39·0°C	> 39·0°C
**Vomiting**	None	1 episode in 24 hours	2-5 episodes in 24 hours	≥ 6 episodes in 24 hours
**Abnormal Crying**	None	< 1 hour	1-3 Hours	> 3 hours
**Drowsiness**	None	Sleepier than usual or less interested in surroundings	Not interested in surroundings or did not wake up for a feed	Sleeping most of the time or difficult to wake up
**Poor feeding**	None	Eating less than normal	Missed 1 or 2 feeds completely	Refused ≥ 3 feeds or refused most feeds
**Irritability**	None	Easily consolable	Requiring increased attention	Inconsolable

Unsolicited Adverse Events

**Mild**	**Moderate**	**Severe**
Easily tolerated, causing minimal discomfort and not interfering with everyday activities	Sufficiently discomforting to interfere with normal everyday activities.	Prevents normal, everyday activities and would necessitate the administration of corrective therapy

Supplementary table 2. Adverse events reported in participants during the study

**n participants [number of events]**<br>**% (95% CI)**	**nOPV2**<br>N = 220	**Placebo**<br>N = 110
**Any Adverse Event (AE)**		
**n [events]**	183 [508]	90 [239]
**% (95% CI)**	83·2% (77·6–87·9)	81·8% (73·3–88·5)
**Any immediate AE**		
**n [events]**	0 [0]	0 [0]
**Any solicited AE**		
**n [events]**	154 [387]	78 [180]
**% (95% CI)**	70·0% (63·5–76·0)	70·9% (61·5–79·2)
**Any severe solicited AE**		
**n [events]**	0 [0]	0 [0]
**Any unsolicited AE**		
**n [events]**	87 [121]	38 [59]
**% (95% CI)**	39·5% (33·0–46·3)	34·5% (25·7–44·2)
**Any severe unsolicited AE**		
**n [events]**	11 [12]	5 [7]
**% (95% CI)**	5·0% (2·5–8·8)	4.5% (1.5–10.3)
**Any serious AE (SAE)**		
**n [events]**	11 [13]	5 [6]
**% (95% CI)**	5·0% (2·5–8·8)	4·5% (1·5–10·3)
**Any AE of special interest (AESI)**		
**n [events]**	0 [0]	0 [0]

Supplementary table 3: Unsolicited adverse events in the two study groups

**System Organ Class Preferred Term**	**nOPV2** (N = 220)		**Placebo** (N = 110)	
	%	Events/ newborn	%	Events/ newborn
**Any Unsolicited AE**	**39·5**	127/87	**34·5**	59/38
**Infections and Infestations**	**35·5**	106/78	**33·6**	57/37
Nasopharyngitis	**10·5**	27/23	**15·5**	20/17
Upper respiratory tract infection	**10·9**	24/24	**10·0**	12/11
Respiratory tract infection	**9·5**	23/21	**8·2**	10/9
Pneumonia	**3·6**	9/8	**3·6**	6/4
Omphalitis	**3·2**	7/7	**1·8**	2/2
Ophthalmia neonatorum	**2·7**	6/6	**0·9**	1/1
Tinea capitis	**1·8**	4/4	**0·9**	1/1
Bronchiolitis	**0·9**	2/2	**0**	0
Oral candidiasis	**0·5**	1/1	**0·9**	1/1
Acarodermatitis	**0·5**	1/1	**0**	0
Furuncle	**0**	0	**0·9**	1/1
Lower respiratory tract infection	**0·5**	1/1	**0**	0
Otitis media acute	**0·5**	1/1	**0**	0
Pneumonia aspiration	**0**	0	**0·9**	1/1
Urinary tract infection	**0**	0	**0·9**	1/1
Varicella	**0**	0	**0·9**	1/1
**Gastrointestinal disorders**	**2·7**	6/6	**0·9**	1/1
Diarrhoea	**0·9**	2/2	**0·9**	1/1
Abdominal distension	**0·9**	2/2	**0**	0
Umbilical hernia	**0·5**	1/1	**0**	0
Vomiting	**0·5**	1/1	**0**	0
**General disorders and administration site conditions**	**2·7**	6/6	**0·9**	1/1
Pyrexia	**2·7**	6/6	**0**	0
Swelling	**0**	0	**0·9**	1/1
**Skin and subcutaneous tissue disorders**	**1·4**	3/3	**0**	0
Dermatitis contact	**0·9**	2/2	**0**	0
Dermatitis atopic	**0·5**	1/1	**0**	0

Supplementary table 4: GMTs of polio neutralising antibodies

Geometric mean titres (GMT) of neutralising antibodies against the three poliovirus types in nOPV2 and placebo groups at baseline, 4 and 8 weeks (Per protocol set).

		nOPV2	Placebo
	**Type 1**		
**Baseline**			
	**GMT**	**29·3**	**39·8**
	(95% CI)	(22·1–38·7)	(27·1–58·5)
**4 weeks**			
	**GMT**	**10·3**	**10·8**
	(95% CI)	(7·5–14·0)	(7·0–16·5)
**8 weeks**			
	**GMT**	**4·4**	**2·9**
	(95% CI)	(3·1–6·1)	(1·5–5·8)
	**Type 2**		
**Baseline**			
	**GMT**	**56·5**	**81·3**
	(95% CI)	(46·4–68·9)	(58·2–113·5)
**4 weeks**			
	**GMT**	**146·8**	**25·8**
	(95% CI)	(107·5–200·6)	(18·3–36·5)
**8 weeks**			
	GMT	**1180**	**10·4**
	(95% CI)	(895·3–1554)	(6·8–16·0)
	**Type 3**		
**Baseline**			
	**GMT**	**15·1**	**11·8**
	(95% CI)	(12·1–18·8)	(7·7–18·3)
**4 weeks**			
	**GMT**	**5·7**	**3·1**
	(95% CI)	(4·5–7·3)	(1·6–6·0)
**8 weeks**			
	**GMT**	**2·5**	**1·6**
	(95% CI)	(1·7–3·5)	(0·7–3·6)
